# Supplementary material for: Are carbon emissions trading and green financial instruments synergistic? -Comprehensive quantitative research based on content analysis
Source: PLoS One. 2024 Mar 7;19(3):e0298601. doi: 10.1371/journal.pone.0298601 (PMC10919601; doi:10.1371/journal.pone.0298601)
Supplement: S1 Data — (ZIP) [file pone.0298601.s001.zip › Data/地级市Z_F工作报告.zip!/─┌├╔╣┼/╬┌║ú╩╨/└┤╫╘╣1⁄2╓┌║┼í╛┬φ┐╦╩2╛▌═°í┐_2012.docx]

各位代表：

我代表市人民政府向大会作工作报告，请予审议，并请各位政协委员和列席会议的同志提出意见。

一、2011年工作回顾

2011年是我市实施“一个中心、两个转型”战略取得明显成效的一年。一年来，在自治区党委、政府和市委的正确领导下，在市人大、政协的支持、监督下，我们深入贯彻落实科学发展观，团结带领全市各族人民，凝心聚力，开拓进取，扎实工作，圆满完成了市七届人大四次会议确定的目标任务，实现了“十二五”的良好开局。预计地区生产总值完成488亿元，按可比价计算增长18%。地方财政总收入完成83亿元，增长22%。固定资产投资完成286亿元，增长20%。社会消费品零售总额完成85亿元，增长18%。城镇居民人均可支配收入达到22000元，增长12%；农区居民人均纯收入达到10700元，增长16%。

产业转型步伐加快，工业经济保持快速增长。预计第二产业增加值完成358亿元，按可比价计算增长19%；155户规模以上工业企业增加值增长21%，增速居全区第3位。工业固定资产投资预计完成154亿元，增长12%。主导产业不断发展壮大，煤焦化、氯碱化工两大基地规模优势进一步显现，广纳、榕鑫、黄河工贸二期、西部煤化等一批百万吨级焦化项目实现投产，焦炭产能达到1600万吨，PVC产量占自治区比重达到62%。加快推进煤炭资源整合，煤炭企业兼并重组全面启动。充分利用周边地区煤炭资源，全年调入煤炭1680万吨，在原煤只增产5%的情况下，洗煤、焦炭分别增产31%和30%。建材、钢铁冶金产业进一步做强，黄河工贸200万吨水泥、利康1万吨氰尿酸和3000吨PVC助剂项目建成投产，蓝星低辐射镀膜玻璃生产线项目进展顺利。包钢万腾节能环保技改项目一期基本完成，已具备100万吨生铁、100万吨钢和60万吨轧材产能；隆兴佰鸿具备6万件LED照明设备产能。产业延伸升级步伐加快，华油焦炉煤气综合利用项目一期、东药乌海化工丙炔醇、盛远塑胶8万吨PVC软制品项目快速推进，东磁光大6000吨多晶硅、东源科技1,4—丁二醇项目前期工作已经完成。非煤、非资源等接续替代产业和战略新兴产业发展迅速，陕汽重卡一期、冀东卡车改装、恒业成一期15万吨有机硅项目进展顺利。认真落实自治区沿黄沿线经济带规划，“双百亿工程”扎实推进，乌海经济开发区营业收入达到500亿元，增幅列自治区第5位。神华乌海能源、源通两户企业营业收入超百亿元。产值超30亿元工业企业达到5户，10亿元以上企业达到16户，亿元以上企业占规模以上企业总户数的60%。乌海经济开发区南区总体规划编制形成初步成果。创新型城市建设深入推进，制定出台鼓励科技创新政策措施，设立3000万元专项资金用于支持工业结构调整和高新技术产业发展，投入550万元用于科技奖励，培育国家级高新技术企业2家，建成自治区级企业研发中心8家，天宇集团与中科院联合研发中心成立，我市被评为全国科技进步先进市。资金争取和招商引资工作成效明显，全年争取上级各类资金26亿元，实施招商引资项目141个，其中产业转型项目占90%；引进到位资金203亿元。我市被成功列为国家第三批资源型城市转型试点。

投资规模不断扩大，第三产业发展后劲持续增强。第三产业固定资产投资完成128亿元，增长33%，占全市固定资产投资的45%。预计第三产业增加值完成125.5亿元，按可比价计算增长11%。服务业发展规划编制完成，制定出台物流业、旅游业等扶持政策。物流业发展步伐加快，乌海焦炭产品交易中心、海勃湾铁路物流园投入运营，广东塑料交易所西部交割仓、如意俊安等物流项目进展顺利。乌海车务段货物发送量突破3000万吨，“黑货”、“白货”运输比例为55：45，“白货”比重进一步提高。商贸服务业快速发展，通用时代广场、中山大厦、乌达谊君半岛国际酒店等项目顺利推进。房地产开发完成投资51亿元，商品房新开工面积244万平方米。金融业加快发展，君正集团成功发行A股并在上海交易所挂牌上市，乌海化工上市工作快速推进。交通银行在我市设立分行，各类银行、保险、证券、小额贷款公司等金融机构达到58家。全市存款余额达480亿元，增长17%；各项贷款余额410亿元，增长18%，其中市外银行贷款余额突破90亿元。旅游业发展迅速，甘德尔山生态文明景区等重点项目加快推进，预计全年游客接待人数达到105万人次，旅游业总收入达到11亿元，增长22%。

城市服务功能日臻完善，人居环境明显改观。城市总体规划（2011—2030年）通过自治区批准，市级土地利用总体规划编制完成，初步完成环乌海湖概念性规划，滨河二期、三期和海北新城规划方案，三区及三区旧城改造控制性详细规划编制工作加快推进。城市建设力度不断加大，全年基础设施建设投入达到80亿元。黄河海勃湾水利枢纽工程进展顺利，基础处理全面完成，征地拆迁完成90%。滨河一期基础设施建设进一步完善提高，甘德尔河改造工程、滨河公园等重点项目进展顺利，滨河二期房屋土地征迁工作有序推进。城市配套功能进一步完善，新建改造新华街等街路22条、23公里。投资6000万元新建大型隔压换热站，建成燃气锅炉热源厂1座、换热站15个，集中供热面积达到1638万平方米，供热质量、供热安全进一步好转。新建配水厂1座、水源井22眼，铺设给排水管网69公里，新增供气管网47公里、燃气用户达到7.4万户，完成单体建筑及景观带亮化97处。城市更新改造步伐加快，实施房屋征迁项目50项，完成征迁3712户、建筑面积51万平方米。立体交通网络进一步健全，三区快速通道、京藏高速乌海互通立交桥、海南黄河路立交桥建成通车，110国道新麻段改造全面完成，海南至乌达重载一级公路加快推进。乌海机场航线航班不断增加，全年旅客吞吐量突破23万人次。制定出台加强社区物业管理政策措施，加大对物业企业的补贴扶持力度，物业服务水平有所提高。全国绿化模范城市和国家园林城市创建工作加快推进，实施白独贵湾、110国道、机场路、林荫大道、乌达西山、乌兰淖尔湖、海南环城绿化等28项重点绿化工程，积极探索沉陷区、棚户区生态植被恢复新模式，共完成造林面积5万亩。实施海南恩格尔河、乌达巴音赛河二期、公铁立交桥等城市绿化美化工程，全年新增绿地面积204万平方米，人均公园绿地面积达到12平方米，绿化覆盖率达到35.3%。加强林地林木资源保护，林木成活率稳步提高。加快淘汰落后产能，淘汰电石21万吨、炼铁36万吨、铁合金13万吨、玻璃390万重量箱，单位GDP能耗下降3%。实施减排项目100项，二氧化硫、化学需氧量预计分别净削减1.5%以上。大力实施“蓝天工程”和重点行业达标整治等环保工程，中心城区空气质量优良天数达到291天。

政策扶持力度加大，城乡一体化发展成效显著。制定实施就业帮扶、子女教育、社保补贴等一系列政策措施，市、区政府投入15亿元用于库区移民和城乡一体化补贴。建设库区转移居民安置楼55栋、3000户，转移库区居民7500余人。加快农区土地流转，推进规模经营，实现土地流转1815亩。葡萄、蔬菜、花卉苗木优势产业进一步发展壮大，汉森等一批龙头企业竞争力不断增强，销售收入百万元以上加工企业达到6户，实现销售收入3.5亿元，增长20%。农业中介组织快速发展，生产经营组织化程度日益提高，蔬菜、瓜果、农机等各类合作经济组织达到21个。全力实施“菜篮子”工程，蔬菜种植面积达到8500亩，35个蔬菜直销店建成运营，蔬菜直供、直销渠道进一步畅通。认真落实各项惠农政策，发放各类补贴资金1758万元。预计第一产业增加值完成4.5亿元，按可比价计算增长5.5%。

民生投入力度空前，人民生活水平稳步提升。全市用于民生和社会事业的财政支出达到49亿元，占地方财政支出总额的70%。年初承诺为民所办十件实事全面完成。大力推进创业带动就业，投入运行创业孵化基地6个，发放小额担保贷款7000万元，成功创业750人，城镇新增就业9600人，城镇登记失业率控制在4.3%以内。高职、中职毕业生就业率达90%以上。出台城乡居民增收意见，最低工资标准经过两次调整，由每月680元提高到1050元。保障性住房建设进展顺利，开工建设各类保障性住房20342套，综合开工率108%，超额完成自治区下达的目标任务，5844户煤矿棚户区居民迁入新居。社会保障水平进一步提高，妥善解决库区移民、滨河二期征迁户养老保险参保问题；帮助1463名原“五七工”参加城镇企业职工养老保险；企业退休人员平均养老金标准每月提高195元，达到1367元。在居民基本医疗保险住院统筹的基础上启动实施普通门诊统筹，实行异地就医结算，将居民医疗保险筹资标准提高到320元，其中财政补助标准由120元提高到269元；帮助3820名关闭破产企业退休人员参加职工医疗保险。全市机关事业单位职工全部纳入生育保险统筹；工伤、失业保险覆盖面进一步扩大。社会救助力度不断加大，儿童福利院、流浪未成年人救助保护中心等项目投入使用。最低生活保障标准由2010年的360元提高到400元，继续保持自治区前列。五保对象集中和分散供养标准每人每年分别提高到6500元和4300元，孤儿集中供养和分散供养标准每人每月分别提高到1000元和800元，均居自治区首位。进一步提高退役士兵安置自谋职业补助金和优抚对象抚恤标准，居自治区前列。60岁以上低收入老年人优待金由每人每年36元增加到100元。积极实施物价稳控措施，为全市低保户和困难群众发放生活补贴和临时价格补贴资金791万元，受益人员达4.7万人次。对涉及房屋征迁的残疾人家庭额外给予不低于2万元的补贴，271户残疾人家庭受益。启动“听力无障碍市”创建，完成重度听力障碍儿童病源筛查工作。发放贫困大学生救助金和市长助学金181万元，资助564名贫困学生顺利入学。继续实施“一杯奶”生育关怀行动，4315名孕期妇女受益。独生子女父母奖励费标准每月提高20元。政府主导扶持企业实施“放心早餐”工程，建成32个经营网点。海南公交分公司组建完成并开始运营；投入2300万元购置公交车50辆，增加公交线路，降低票价，群众出行更加方便快捷。

社会事业快速发展。制定出台中长期教育改革与发展规划纲要。校安工程第一阶段任务超额完成，累计投资7.2亿元， 新建31所学校、建筑面积30万平方米。市十中报告厅、教学楼和蒙古族中学教学楼、服务楼建成使用。财政安排1500万元设立学前教育发展专项资金，开工建设标准化幼儿园8所，建成3所。投入4680万元用于职业教育基础设施建设，职业技术学校被列为国家级中等职业教育改革发展示范学校。实行公办普通高中、民族教育、中等职业教育全免费政策，受益人员达2.6万人次，实现十二年免费教育。推行名校名校长名师“三名”工程，教育教学质量不断提高。医药卫生体制改革稳步推进，基层医疗卫生机构和公立医院改革取得实效。加强公共卫生基础设施建设，市人民医院内科楼主体完工，妇儿医疗预防保健中心投入使用。连续4次被评为全国无偿献血先进城市。文化体育基础设施进一步完善，建设文化站20个，草原书屋达到54个，新增各类健身路径80套，海勃湾青少年宫投入使用，书法产业创意园区、乌海博物院、书法艺术中心和岱山葡萄酒博物馆进展顺利。通过以奖代补投入300万元扶持群众性文化活动。成功举办第二届国际书法产业博览会、全国“市长杯”乒乓球赛和首届全民健身运动会。书法城建设深入推进，我市被命名为“中国硬笔书法名城”，财政出资选派中青年书法人才外出进修培训，免费为市民开通书画频道。文化产业繁荣发展，书院、书画廊、奇石、仿古家具店铺达到50余家，年经营总收入超亿元。投资5000多万元完成广播电视设备升级和乌达、海南有线网络数字化改造工程，率先在全国实现农区居民广播电视“户户通”。

社会管理创新深入推进，和谐社会建设开创新局面。民主法制建设稳步推进，自觉接受人大法律监督、工作监督和政协民主监督，办理市人大代表议案、建议126件、政协委员提案146件。认真听取各民主党派、工商联、人民团体和社会各界意见。服务型政府建设不断深化，政务服务大厅、电子监察平台和公共资源交易中心的作用得到充分发挥，行政许可审批事项和行政事业性收费项目全部进驻政务服务中心办理，清理行政许可审批事项109项，取消和停收行政事业性收费项目117项。强化对政府投资项目、预算执行情况和民生领域资金审计工作，保障了资金的良好运行。加强基层民主建设，“五五”普法工作圆满完成，“六五”普法全面启动，我市被评为“全国法制教育先进城市”。深入开展群众性精神文明创建活动，成功举办全市第三届精神文明建设经验交流会。完善社区基础设施，新改扩建标准化社区综合服务中心23个。推行街道管理服务体制改革试点，基层社会管理和服务水平不断提高。开展社会治安集中打击整治“夏季攻势”行动和 “保平安、促和谐”百日专项行动，社会公共安全保障得到进一步强化。加强安全生产监管，严格执行“党政同责”、“一岗双责”等各项安全生产制度，强化重大危险源监控体系建设，加强危险化学品、非煤矿山、道路交通等重点领域安全监管，4个新建特勤消防站投入使用。深入推进“科技防控城市”建设，重点区域监控系统全部建成使用。认真落实信访工作责任制，及时排查化解社会矛盾，一批关系群众切身利益的信访问题得到妥善解决。加强食品药品安全监管，三区食品药品监管部门实行属地管理，国家药品安全示范市创建工作全面启动。人口自然增长率控制在6‰以内，人口和计划生育工作继续保持全区前列。连续六次被评为自治区“双拥模范城”。防震减灾工作进一步加强，活断层探测项目正式启动。国防建设、民兵预备役、档案史志、侨务外事、人防、气象、新闻出版、无线电管理、红十字和老龄事业等工作均取得新成绩。

各位代表，过去一年取得的成绩，是自治区党委、政府和市委正确领导的结果，是全市各族人民共同团结奋斗的结果，在此，我代表市人民政府，向在各个领域和岗位上辛勤工作的各族干部群众，向给予我们大力支持的人大代表、政协委员，各民主党派、工商联和社会各界人士，向驻市部队、武警消防官兵、公安干警和各方面朋友，表示崇高的敬意和衷心的感谢！

在看到成绩的同时，我们也清醒地认识到发展中存在一些问题，主要是：产业发展不充分，产业结构单一，产品初级化、低端化特征明显，科技支撑和自主创新能力不强，抵御市场风险能力较弱；第三产业比重偏低、层次不高，发展相对滞后；固定资产投资后劲不足，对经济增长起决定作用的重大项目还比较少；城市服务功能不完善，基础设施建设与城市提质扩容、人口集聚不相适应，城市管理有待加强；生态建设和环境保护任务艰巨，节能减排和安全生产压力较大，部分产业能耗和污染物排放偏高，粉尘污染较重，空气质量有待进一步提高；城乡居民收入滞后于经济发展，部分低收入群体生活比较困难，教育、卫生、文化等优质公共服务资源总量不足、分布不均；政府自身建设需要进一步加强，一些工作人员的思想观念、工作作风还不适应新形势、新任务的要求。对于这些问题，我们将高度重视，认真研究，在今后的工作中切实加以解决。

二、2012年工作安排

2012年市政府总体工作思路是：以科学发展观为统领，认真贯彻落实中央、自治区经济工作会议、市第六次党代会和市委六届三次全委会议精神，坚持富民与强市并重、富民优先，深入实施“一个中心、两个转型、三个提升”发展战略，全面落实“城市提质年”各项工作，推动经济社会又好又快发展，为建设自治区西部区域中心城市奠定坚实基础。

主要预期目标是：地区生产总值完成590亿元，增长21%；地方财政总收入完成101亿元，增长22%；固定资产投资完成340亿元，增长20%；社会消费品零售总额完成102亿元，增长20%；城镇居民人均可支配收入达到25300元，增长15%；农区居民人均纯收入达到12480元，增长17%；节能减排完成自治区控制性指标；城镇登记失业率控制在4.3%以内；人口自然增长率控制在6‰以内。

实现上述目标，要重点做好八个方面的工作：

（一）做大总量，调优结构，加快推动经济转型

做强做优支柱产业。认真抓好国务院出台的《关于进一步促进内蒙古经济社会又好又快发展的若干意见》贯彻落实，千方百计扩大投资规模，工业固定资产投资力争超200亿元。大力实施“双百亿工程”，深入推进“一城两基地”建设。全市产值超百亿元的企业力争达到4户以上。加快华信、宏阳、远大、美方二期等百万吨级焦化项目建设，重点抓好中联化工一期20万吨PVC、懿峰工贸双50万吨PVC和烧碱项目建设。积极稳妥推进煤炭资源整合，提高市外原煤利用比重。推动产业延伸升级，加快推进华油焦炉煤气综合利用项目二期、神华乌海能源硝铵、东药乌海化工丙炔醇、东源科技1,4—丁二醇项目建设，突出抓好三木集团精细化工、百合科莱恩二萘酚项目。推动建材、钢铁冶金产业升级换代，完成蓝星集团搬迁升级改造，加快赛马二期、乌海化工电石渣制水泥和黄河工贸矿渣制水泥项目建设。支持包钢万腾全面达产并发展下游产业，开工建设明海锆业氧化锆、恒兴冶炼镍铁项目。力争完成华宜自备电厂、京海煤矸石电厂二期报批工作，争取年内开工建设。积极推进乌达高新技术园区动力车间项目，抓好乌海热电三期、国电电力等项目前期工作。

培育发展接续替代产业和新兴产业。加快推进陕汽重卡二期、唐山神州机械、新通领特种电缆等装备制造业项目建设，积极推动盛远塑胶8万吨PVC软制品、恒业成有机硅、东磁光大多晶硅、隆兴佰鸿二期等项目，重点抓好抽水蓄能电站、三花科技碟式太阳能电站、蓝星集团非晶硅薄膜电池、维斯通管业、斯凯夫高档涂料等新能源、新材料项目建设。非煤产业占工业增加值比重达到35%以上。

大力提升园区承载能力。市政府启动并加快乌海经济开发区南区规划建设，编制完成南区控制性详细规划，实施道路、供水、输变电等基础设施工程，年内具备项目入驻条件。南区作为我市的低碳产业园区，力争获得自治区级以上批复。制定出台发展“飞地经济”政策措施，在全市范围内合理布局项目，建立统一的产业准入、项目投资、权责对等、利益共享等方面政策体系。完善南区管理，三区环保、安监、工商、税务等部门向南区延伸管理职能。全力推进国家承接产业转移示范区申报工作，积极承接发达地区先进生产力转移。贯彻落实沿黄沿线经济带规划，编制完成三个工业集中区产业发展规划，加快推进乌海经济开发区申报国家级开发区工作。建立完善资源城市转型发展以项目为中心的政策体系，主动加强与周边地区沟通协作，在推动区域经济整体转型发展中发挥主导作用。坚持节约集约用地，结合矿区搬迁改造，盘活工矿区、采煤沉陷区闲置土地。全力做好招商引资工作，创新招商方式，力争全年引进到位资金230亿元。

全面提高企业核心竞争力。加强宏观经济运行监测分析，认真落实技术改造、信贷支持、就业、减免收费等方面优惠政策，主动帮助协调解决企业经营发展过程中存在的困难和问题，促使企业尽快提质增效。全面实施“千户中小企业成长工程”，编制完成中小企业发展中长期规划，积极引导小微企业围绕优势特色产业和骨干企业协作配套发展。深入推进“两化融合”，制定出台科技示范项目资金扶持办法，引导企业加大科技投入，进一步提高全社会研究与开发经费占GDP比重。依靠科技创新改造提升传统产业，新培育自治区级企业研发中心2家。大力推进质量兴市战略，引导企业加强质量管理和标准化体系建设，努力争创中国名牌产品和驰名商标。

（二）完善功能，提升品位，加快推动城市转型

高起点编制城市规划。组织实施好乌海及周边地区城镇总体规划和乌海市城市总体规划。加快编制环乌海湖规划，完成三区控制性详细规划，推进滨河二期、三期、西区、海北新城等总规、控制性详细规划编制工作。做好城市重要节点和主要街路的城市设计工作。

高标准推进基础设施建设。力争政府主导的基础设施投入超过100亿元。加快黄河海勃湾水利枢纽工程建设进度，力争年内具备蓄水条件，开工建设库区东侧护岸景观工程。加快滨河一期中心商业区和居民生活服务区建设，建成神华研发中心综合楼、中行营业大楼等工程。加快滨河二期基础设施建设，基本完成滨河二期主次干道和市政管网建设，开工建设滨河区配水厂等公用配套设施。做好城市水系规划建设，完成甘德尔河水系设计和景观二期工程，启动摩尔沟水系建设。加快旧城区改造，完善旧城区供热、供气、给排水等市政公用基础设施，年内新增集中供热面积240万平方米，新建配水厂2座，铺设给排水管网230公里。加快老旧小区地下管网等公用基础设施改造，提高老旧小区物业管理水平。加大城市棚户区和平房区房屋拆迁改造力度，改善居民居住条件。大力实施园林绿化工程，完成植物园改造，建成铁路沿线海勃湾段葡萄主题公园，基本完成机场路、海勃湾至海南城际快速通道两侧绿化带建设，开展城区重要节点、道路、庭院绿化建设。全面推进城市路网建设，新建改造海勃湾建设北路、乌达巴音赛街、海南巴音陶亥街等街路27条，建成海南至乌达重载公路、109国道和217省道连接线一级公路，开工建设跨乌海湖大桥、110国道黄河特大桥、西来峰至苦水沟一级公路，力争开工扩建新地至西来峰运煤一级公路，启动乌达环城公路规划设计和项目开工，抓好东乌与包兰连接线、南区至上海庙新建铁路和南区铁路专用线等重点铁路建设工程，推进荣乌高速公路乌海段工程，构建快捷畅通的交通体系。

高水平加强城市管理。大力推行城市精细化、人性化管理，深入开展市容卫生环境综合整治，增加环卫设施，新建垃圾转运站4座、环卫工人休息室18间、公厕22座。加强城市管理综合执法，依法打击私搭乱建等违法行为。强化交通环境整治，启动建设全市智能交通管理系统，规范城区停车管理。进一步加强信息工作整合力度，继续推动“数字乌海”建设，完善全市地籍信息管理系统。深化公交体制改革，建立健全管理、考核、评价机制，组建完成乌达公交分公司并投入运营，提升公共交通服务水平。加强和规范物业管理，培育大型物业管理企业，加大扶持和补贴力度，全面提升物业管理水平。加强市民素质教育，动员全社会自觉维护城市形象，努力营造整洁优美的城市环境。

（三）提高比重，提升层次，促进第三产业提质增效

完善政策支撑体系。全面实施服务业发展规划，制定出台扶持政策，设立专项引导资金，积极推进服务业综合改革试点区申报工作，努力推动第三产业发展提速、比重提高和竞争力提升。

大力发展生产性服务业。编制物流业和空港物流发展规划，积极发展第三方物流，重点抓好乌海西物流园建设，推动实施乌吉铁路电气化改造工程，协调尽快打通乌海至策克运煤铁路通道。支持同洲物流园、焦炭产品交易中心做大做强，积极推进如意俊安二期、广东塑料交易所西部交割仓等项目建设，确保早日投入运营。加快乌海海关设立申报工作。

繁荣发展生活性服务业。完善滨河新区和西区商业网点规划布局，建成通用时代广场，加快人民路和新华街城市商业中心改造、麦当劳餐饮连锁店等项目建设，开工建设维多利财富广场、九龙国际广场、金鼎综合商场等项目，着力引进沃尔玛连锁超市、开元酒店等一批知名品牌项目。

培育壮大现代服务业。积极引进包商银行、鄂尔多斯银行、光大银行、浦发银行在我市设立分支机构。扎实推进16亿元城投债券发行，争取引入1家基金管理公司，推动乌海化工并购上市。加快发展保险、信托、证券等金融服务业，加强融资平台建设管理，切实防范和化解金融风险。加快发展商务会展、服务外包、中介资讯等相关行业，大力开发便民服务、家政服务等项目。强化房地产市场监管，推进房地产业有序健康发展。全面完成市第一粮库退城进郊，推动中石油乌海分公司油库搬迁和中石化乌海分公司油库建设。

积极发展旅游业。编制完成环乌海湖、甘德尔山景区等旅游规划，加快甘德尔山生态文明景区和龙游湾生态旅游区项目建设，启动胡杨岛保护与开发项目，做好环乌海湖健身休闲项目前期工作。力争全年接待游客118万人次，旅游总收入增长22%以上。

（四）落实政策，推进转移，加快提高城乡一体化发展水平

坚持“转移、减少、集中、富裕”的发展思路，依托经济开发区南区建设，统筹推进巴音陶亥镇城乡一体化和新农区建设，全力做好库区、园区的土地房屋征迁工作，妥善解决搬迁居民的生产生活问题，推动农区居民向城市有序流动。通过信贷贴息、社保优惠、岗位补贴等扶持政策，多渠道安置农区劳动力就业，提高农区居民非农收入。加大农区道路建设力度，实现全市村村通沥青混凝土公路。加快农区土地流转，推进土地规模化经营。加大葡萄、蔬菜、花卉苗木产业扶持力度，扶持发展设施农业，支持市农业科技示范园、汉森、云飞等一批龙头企业尽快做大做强。充分利用闲置盐碱洼地和乌海湖，发展规模化、标准化水产养殖。全力实施“菜篮子”工程，制定出台蔬菜产业发展专项规划，加强本地蔬菜产销对接，建立市外蔬菜基地，建设农副产品信息化系统，加快仓储物流建设。新增葡萄种植面积3000亩，新增蔬菜种植面积4000亩，其中新增保护地1500亩。重点实施景裕园、田野、山林公司无公害蔬菜项目，抓好乌达农副产品集散中心和冷链物流配送中心建设，新建农产品质量安全检验检测站，推进农副产品及蔬菜直销店建设，扩大无公害、绿色农产品认证覆盖面。

（五）突出特色，优化服务，着力增强文化软实力

大力发展文化事业。编制文化改革发展规划及“十二五”文化发展规划纲要。加强文化基础设施建设，建成乌海博物院、书法艺术中心和一批露天文化广场、小剧场，开工建设市汽车电影城，启动广播电视大厦和多功能发射塔建设前期工作，继续实施广播电视系统传输技术装备一体化集成改造。大力实施文化惠民工程，着力打造广场文化节、全市文化艺术节等文化活动品牌，广泛开展群众性文化活动，丰富人民群众文化生活。统筹推进文化经营管理人才、公共文化服务人才、基层文化人才等各类文化人才队伍建设，提高文艺创作水平，尽快推出书法文化影视作品和舞台精品剧。加强历史文化遗产保护，争取满巴拉僧庙列入自治区非物质文化遗产名录。

培育发展文化产业。制定促进文化产业发展的意见，科学策划和精心打造具有一定影响力的节庆品牌，鼓励和规范市场化运作和企业、协会承办，努力提高城市的知名度。规划建设书法文化创意产业园区，进一步扩大和提升“中国书法城·乌海”品牌影响力，带动文房四宝、书画奇石、文博会展、文化创意、动漫影视等产业发展。推进文化与旅游深度融合，依托环乌海湖和甘德尔山生态文明景区开发建设，促进移民文化、书法文化、草原文化、黄河文化融合发展。

（六）强化措施，落实责任，努力提升地区环境保护、生态建设、安全生产水平

强力推进节能减排。坚决落实节能减排目标责任制，制定 “十二五”节能减排综合性工作方案，全面推行固定资产投资项目节能评估审查制，大力淘汰落后产能，全年淘汰铁合金7万吨、水泥64万吨、石灰竖窑400孔。积极推进建筑节能改造工程，完成改造面积50万平方米。严格执行项目审批污染物排放指标前置制度，推进污染物防治由末端治理向源头控制转变。加快污水、垃圾处理等基础设施建设，完成市生活污水处理厂和海南污水处理厂扩建工程，开工搬迁乌达生活污水处理厂扩建工程，建成海勃湾餐厨垃圾处理、乌达和海南生活垃圾无害化处理项目。加强水资源规划管理，大力推行节水工程，科学开发、严格利用地下水资源，稳步推进水权置换。积极推行清洁生产，大力发展循环经济。提高粉煤灰、煤矸石等工业“三废”资源综合利用效率，抓好蓝益垃圾发电、江苏双良余热利用等项目建设。编制循环经济发展规划，积极做好国家级循环经济试点城市申报工作，全市循环经济示范企业达到10户。

大力加强环境保护。抓好矿产资源开发综合整治，开展露天开采、白灰生产、洗煤、化工、建材、砂石料等行业专项治理，重点治理工业烟尘、粉尘、扬尘污染，积极争取国家级矿山地质环境治理项目。加强地下水保护和水质监测，启动实施农区饮水安全工程，保障居民饮水安全。强化黄河水污染防治，确保水质达到三类水环境功能要求。加强环境和节能监测监察能力建设，进一步完善重点污染源自动监测系统。强化环境执法监督，推动区域环境联防联控，继续深入实施环境保护六大工程，中心城区空气质量二级以上天数达到295天，乌达、海南城区均达到230天。

全力推进生态建设。继续实施天然林保护、三北防护林等国家重点林业项目，加强采空区、沉陷区生态恢复，全力抓好环乌海湖绿化项目。加大投入力度，推动乌阿边界乌兰布和沙漠锁边林项目建设。投资3.8亿元，重点实施人民路南出口、京藏高速海勃湾出口北侧、110国道等26项重点城市绿化工程，年内完成绿化造林面积5.9万亩，人均公园绿地面积达到12.3平方米。

严格落实安全生产“党政同责”、“一岗双责”责任制，进一步健全安全生产体制机制，加快安全生产风险评估预警、应急处置、事故救援体系建设，提高安全生产保障能力。突出抓好煤矿、非煤矿山、交通运输、危险化学品等重点领域的安全监管，严防各类安全生产事故发生。

（七）加大投入，富民惠民，全面提升公共服务水平

着力加强以保障和改善民生为重点的社会建设，今年财政支出的70%以上用于民生和社会事业，不断满足群众对社会公共服务的需求。

积极推进创业带动就业。全力做好就业工作，城镇新增就业9000人，实现失业人员再就业6000人；开发公益性岗位1500个，优先解决库区移民、巴音陶亥镇农区转移居民就业问题。突出抓好高校毕业生创业就业，建立人社部门、就业、教育、团委等多部门促进就业联动机制，将高校毕业生纳入失业登记制度范围，鼓励中小企业吸纳高校毕业生就业，共同解决好高校毕业生就业问题。完善落实小额担保贷款、财政贴息、税费减免、技能培训等鼓励政策，发放小额贷款6500万元，年内培训9500人次。

切实提高城乡居民收入。突出富民优先，逐步缩小城乡居民收入差距，完成乌达城乡居民收入并轨。引导企业完善职工工资收入正常增长机制，健全工资集体协商机制和工资支付保障机制。落实国家提高艰苦边远地区津贴、节假日补贴政策，逐步提高最低工资标准，推进事业单位绩效工资制度改革。畅通流通渠道，减少中间环节，降低物价总水平。进一步加大扶持力度，在增值税、营业税起征点调整为2万元的基础上，继续落实减轻中小微利企业、个体工商户和创业人员税费负担的各项政策，年内减免增值税、营业税和企业所得税4300万元，确保9600户企业、个体工商户受益。强化劳动保障执法监察，切实维护劳动者合法权益。

完善社会保障体系。强化定点医疗机构监管，推行医保机构与定点医院总额预算结算办法。将用人单位职工、关闭破产企业退休人员全部纳入医疗保险，推进医疗保险由制度全覆盖向人口全覆盖转变。扩大享受工伤保险范围，将“老工伤”人员全部纳入工伤保险统筹管理。推进社会保障信息化建设。加大弱势群体救助力度，切实落实低收入群体补贴与物价上涨联动机制。完善城乡医疗救助体系，加大对困难群体的医疗救助力度，重点对因病致贫、因病返贫家庭进行救助，切实改善工病亡家属、残疾人、低保边缘户等弱势群体的生活条件。关心老年人生活，高标准新建市老年养护服务中心和15个社区老年人日间照料中心，扩建市宜和老年公寓；为60岁以上低收入老年人发放高龄津贴，积极探索和推进社区居家养老模式，推动社会化养老事业发展。加快发展公共服务和社会福利事业，新建改造5个社区综合服务中心和市救灾物资储备库。加快保障性住房建设，新建各类棚户区改造住房7591套、公共租赁住房800套，着力解决好中低收入家庭和困难群众住房问题。关心支持残疾人事业，继续实施“白内障无障碍市”和“听力无障碍市”创建工作，免费为白内障患者实施复明手术、为适龄聋儿实施人工耳蜗植入手术、为残疾人提供辅助器具800件。免费开展19项孕前优生健康检查项目，进一步提高出生人口素质。扶持实施“放心早餐”工程，建设放心早餐网点10个，流动早餐亭100个。

加快发展社会事业。坚持教育优先发展，继续加大投入力度，推动学前教育发展，新建10所标准化幼儿园，解决幼儿入园难问题。狠抓教育教学管理，加大师资队伍培训力度，全面提高教育教学质量。推进科研兴教和“三名”工程，全面推行校长和优秀教师职级制工资，调动学校管理人员和教师队伍积极性。加快中小学信息化建设，提高教育现代化水平。加快发展职业教育，新建1所职业高中，大力培养实用人才和高技能人才。进一步提高中小学生人均公用经费标准和特殊教育学生生活补贴。重视和支持民族教育、特殊教育发展。深化基层医疗机构改革，推行公立医院绩效管理改革，促进基本公共卫生服务均等化。加大医疗卫生基础设施建设力度，开工建设精神卫生中心、职业病防治院、传染病院，高标准规划建设上海第二军医大乌海医院、海勃湾医院等区级医院，新建改造8个社区卫生服务中心。完善突发公共卫生事件应急处置机制，提高重大传染病和疫情防控能力。加大便民健身设施建设力度，为公共场所和居民住宅小区配建100套健身路径，引导鼓励企业等社会力量建设室内体育场馆。广泛开展群众性体育活动，办好第二届全民健身运动会，积极筹办国内外知名体育赛事。加强精神文明建设，继续实施市民素质提升工程，开展文明城市、文明社区、文明行业、文明单位创建活动。

加强和创新社会管理。推进社会管理重心下移，逐步推广政府向社会购买服务，设立社会管理创新专项资金，提高文化体育、交通协管、治安巡逻、社区管理等服务水平。健全信访工作责任制，加大矛盾纠纷排查化解力度，畅通群众利益诉求渠道。大力推进“六五”普法，切实加强人民调解、法律援助和法律服务。深入推进“科技防控城市”建设，严厉防范和打击各类违法犯罪活动，不断增强人民群众的安全感。进一步健全应急管理机制，提高公共安全保障和突发事件处置能力。积极创建国家级药品安全示范市，进一步理顺食品药品监管体制，启动“放心粮油”工程，建设食品安全城市，切实保障群众饮食用药安全。加强基层群众自治组织建设，精心组织全市第八次村（居）委会换届选举工作。进一步做好人口和计划生育、民族宗教、档案史志、双拥优抚、侨务外事、气象、防震减灾、无线电管理等工作。

（八）创新管理，提升效能，建设人民满意政府

大力推行依法行政，认真落实人大及其常委会的决议和决定，自觉接受人大依法监督、政协民主监督，主动听取各民主党派、工商联、无党派人士和各人民团体的意见，高质量办理人大代表建议和政协委员提案，确保依法、公正、高效行使权力、履行职责。创新政务公开方式方法，深入推进政务公开、政府信息公开和公共事务公开，提高政府工作透明度。切实提高行政效能，全面推行“三三制”工作法，继续清理和规范行政许可审批行为，有效减少办事环节，固化工作流程，压缩办结时限。积极探索并联审批、网上审批模式，不断提升市、区、镇（街道）政务服务中心和便民服务中心功能。进一步完善街道办事处财源建设体系和财力运行机制，积极稳妥地推进教育、卫生等事业单位财务由会计核算中心统一管理。着力推进廉政建设，完善政府重大投资项目公示制和责任追究制，加强完善政府采购、招投标管理，严厉打击虚假资质、围标串标、转包挂靠等违法违规行为。全面推行公务卡结算，加强对重点项目、重点行业、重点资金的审计和监管，严肃查处违法违纪案件，进一步营造风清气正、廉洁高效的政务环境。

各位代表，我市正处于转型发展、科学发展、和谐发展的关键时期，前景无限美好，形势催人奋进。让我们在市委的坚强领导下，紧紧团结和依靠全市各族人民，进一步解放思想，奋力拼搏，为加快经济和城市转型、建设自治区西部区域中心城市而努力奋斗！
